# Changes in *FTO* and *IRX3* gene expression in obese and overweight male adolescents undergoing an intensive lifestyle intervention and the role of *FTO* genotype in this interaction

**DOI:** 10.1186/s12967-019-1921-4

**Published:** 2019-05-24

**Authors:** Saeid Doaei, Naser Kalantari, Pantea Izadi, Tuire Salonurmi, Alireza Mosavi Jarrahi, Shahram Rafieifar, Ghasem Azizi Tabesh, Ghazaleh Rahimzadeh, Maryam Gholamalizadeh, Mark O. Goodarzi

**Affiliations:** 1 Research Center of Health and Environment, Guilan University of Medical Sciences, Rasht, Iran; 2 Cancer Research Center, Shahid Beheshti University of Medical Sciences, Tehran, Iran; 3 Department of Community Nutrition, School of Nutrition and Food Sciences, Shahid Beheshti University of Medical Sciences, Tehran, Iran; 4 Department of Medical Genetics, School of Medicine, Tehran University of Medical Sciences, Tehran, Iran; 5 Department of Internal Medicine, Oulu University Hospital and University of Oulu, Oulu, Finland; 6 Faculty of Medical School, Shahid Beheshti University of Medical Sciences, Tehran, Iran; 7 Health Promotion and Education Department, Ministry of Health, Tehran, Iran; 8 Department of Medical Genetics, School of Medicine, Shahid Beheshti University of Medical Sciences, Tehran, Iran; 9 Institute for Intelligent Systems Research and Innovation (IISRI), Deakin University, Geelong Waurn Ponds, Australia; 10 Student Research Committee, Cancer Research Center, Shahid Beheshti University of Medical Sciences, Tehran, Iran; 11 Division of Endocrinology, Diabetes and Metabolism, Department of Medicine, Cedars-Sinai Medical Center, Los Angeles, CA USA

**Keywords:** Obesity, Gene expression, Genotype, *FTO*, *IRX3*

## Abstract

**Background:**

Lifestyle intervention may have a critical effect on the association between genetics and obesity. This study aimed to investigate changes in *FTO* and *IRX3* gene expression in obese and overweight male adolescents undergoing a lifestyle intervention and the role of *FTO* genotype in this interaction.

**Methods:**

This study was a field trial of 62 adolescents from boys’ high schools in Tehran, Iran. Two schools were randomly allocated as the intervention (n = 30) and control (n = 32) schools. The rs9930506 SNP in *FTO* was genotyped at baseline and the level of *FTO* and *IRX3* expression in peripheral blood mononuclear cells (PBMCs). Anthropometric measurements were assessed at baseline and after 18 weeks of intensive lifestyle intervention.

**Results:**

Our results showed that *IRX3* expression in the intervention group was significantly up-regulated compared to baseline (P = 0.007) and compared to the control group (P = 0.011).The intervention group had significantly up-regulated transcripts of *IRX3* only in rs9930506 risk allele carriers of the intervention group compared to risk allele carriers of the control group (P = 0.017). Moreover, our data showed that the FTO expression was up-regulated in AA genotype carriers and down-regulated in AG/GG genotype carriers (P = 0.017).

**Conclusion:**

Lifestyle modification may exert its effects on obesity through changes in the expression level of the *FTO* and *IRX3* genes. However, *FTO* genotype plays a role in the extent of the effect of lifestyle changes on gene expression. Further studies are crucial to have a better understanding of the interaction between lifestyle, genetics and anthropometric measurements.

*Trial registration* This paper reports a comprehensive intervention study (Interactions of Genetics, Lifestyle and Anthropometrics study or IGLA study), which is retrospectively registered in the Iranian Registry of Clinical Trials as IRCT2016020925699N2. Date registered: April 24, 2016. (https://www.irct.ir/searchresult.php?id=25699&number=2)

**Electronic supplementary material:**

The online version of this article (10.1186/s12967-019-1921-4) contains supplementary material, which is available to authorized users.

## Background

Obesity in young people has been dramatically increased in recent years [1]. The prevalence of obesity among young adults in developing countries ranges from 2.3 to 12%, with rates of being overweight as high as 28.8% [2]. Obese people have a greater risk of many chronic diseases such as diabetes, cardiovascular disease, cancer, psychological conditions and mortality [3]. Hence, practical comprehensive interventions are needed to mitigate obesity in young individuals.

Obesity is a multifactorial disorder caused by both genetic and environmental factors [4, 5]. Recent studies reported that obesity is 25–40% heritable [5]. Many genetic loci have been associated with obesity, and the *FTO *locus has the greatest effect size [6]. It is reported that FTO genotype had a strong association with body weight and body composition [6]. This results remained significant after adjustments for calorie intake and physical activity. It seems that the effects of FTO genotype on anthropometric indices is independent from calorie intake and energy expenditure (BMC). However, the exact mechanism of these changes has not been determined yet, but it’s suggested that FTO exert its effects through change the expression level of Iroquois-related homeobox 3 (IRX3) gene. IRX3 is a member of the Iroquois homeobox gene family and plays a role in an early step of neural development. The expression level of this gene in hypothalamus is reported to be related to calorie intake and body composition [7].

On the other hand, environmental factors including dietary intake and physical activity have a critical role in determining body weight and body mass index (BMI) [4]. Recent studies suggest that lifestyle changes can modify the magnitude of effect of genetic predisposition for obesity [7]. For example, over-eating and physical inactivity have increased obesity in recent decades with different mechanisms e.g. increase the level of FTO gene expression [8]. Also the dietary changes can affect the microbiome and therefore change the host methylation which is important in diet-induced obesity [8]. Moreover, people with the risk allele at *FTO* may be more vulnerable to diet-related obesity [9]. Therefore, we need to identify optimal interventions that can reduce the prevalence of obesity through direct (by reducing intake and increasing calorie expenditure) and indirect (through interactions with obesity related genes) mechanisms. This study aimed to investigate the changes in *FTO* and *IRX3* gene expression in obese and overweight male adolescents undergoing an intensive lifestyle intervention and the effect of *FTO* genotype on these changes.

## Methods

### Research context and subject recruitment

The following details are presented in accordance with the CONSORT reporting guidelines for randomized trials of non-pharmacologic treatment (Additional file 1). This study was a field trial and details of the trial have been published elsewhere [10]. In brief, participants were overweight or obese adolescent boys. The inclusion criteria were age 12 to 16 years, students’ willingness to participate in the study, and reaching the puberty stage, BMI ≥  + 1 z-scores, and age 12–16 years. The specific exclusion criteria included: suffering from diseases effective on body weight, treatment with the drugs that effect on body weight, fear of blood sampling, implausible data on BMI or difficulty in finding the veins. To evaluate more accurately the group effect, the a-priori computed sample size of 60 students (30 students in each group) was required. A randomized stratified sampling was used and 540 students in two boys’ high schools (including grades 7–9) of a randomly chosen district of Tehran city (District 5) attended an information session, of which 246 were eligible to participate in the parent trial. Of these, 96 expressed interest in participating in the ancillary study, 84 enrolled and consented to the blood sampling at baseline and week 18, and 62 provided both baseline and week 18 blood samples. Thus, 62 participants were included in the analysis. Two schools were randomly allocated as the intervention (n = 30) and control (n = 32) schools. All measures were taken between morning and noon at baseline and after 18 weeks of intervention.

### Quantitative real-time PCR

At baseline and week 18, fasting blood samples (5 ml) were collected of all students who participated in the study, transferred to EDTA tubes and stored at − 80 °C. Total RNA from peripheral blood mononuclear cells (PBMCs) was subsequently isolated using the GeneAll RNA extraction kit (GeneAll, South Korea), cDNA synthesis was performed using the GeneAll cDNA synthesis kit (GeneAll, South Korea), and gene expression levels were determined using the Optic on real-time PCR detection system (Bio-Rad Laboratories, California). Reactions were carried out in duplicate using SYBR Green Gene Expression Master Mix (Cat. No. 638317; Takara, Japan). Melting curve and gel electrophoresis analysis of the amplification products was used to confirm that the primers amplified only a single product of expected size (data not shown).The HPRT gene was used as the reference gene for normalization, chosen because of its stable expression in blood cells. Quantification of transcripts of interest relative to the internal housekeeping control gene HPRT was performed using the 2^−ΔΔCt^ method and expressed as fold change. Changing *FTO* and *IRX3* expression was evaluated using the REST (Relative Expression Software Tool) software. Data on changes of gene expression were transferred to SPSS software in order to analyze relationships with *FTO* genotype.

### Genotyping

The DNA extraction kit manufactured by GeneAll was used to extract and purify DNA samples. The NanoDrop device (Thermo Scientific, Wilmington, DE, USA) was used to quantify DNA concentration. The optical density (OD) of the samples was measured at a wavelength of 260–280 nm. The quality of the extracted DNA was checked by agarose gel electrophoresis. In brief, genomic DNA was amplified by PCR using the Taq DNA Pol 2X Master Mix Red (Cat. No. A180301; Ampliqon, Denmark). The PCR products were sequenced by GeneAll. The rs9930506 SNP in *FTO* was genotyped in all the subjects and the quality and average length of the sequence library for each sample was assessed using the Chromas software (version 2.33, https://www.Technelysium.com.au/chromas.html).

### Intervention

An 18-week comprehensive lifestyle modification was prescribed to the intervention group. At this level, the personalized diet and physical activity intervention were implemented for each participant. In addition, parents were provided an educational session regarding healthy meals and creating a supportive environment at home for healthy diet and physical activity for adolescent boys. The method of appropriate implementation of diet has been instructed to parents and students through a face-to-face training, followed by booklets and phone calls. A personalized diet for weight management for each participant was adopted. Free healthy snacks were also offered in school days by researchers. Furthermore, a high-intensity interval training was carried out for improving the physical activity at the schools. In this method, students were involved in high-intensity exercise for a minimum of 30 min 3 days per week. Moreover, three education sessions focused on healthy lifestyle were held. The control subjects were allowed to continue their usual daily activities and diet. Details of the intervention was published elsewhere [10].

### Assessment of other variables

Usual dietary intakes of participants were examined by a validated 168-item semi-quantitative FFQ. The FFQ consisted of 168 food items with standard portion sizes commonly consumed by Iranian people. Daily intakes of food groups and calorie for each person were analyzed. The International Physical Activity Questionnaire (IPAQ) was used for measuring physical activity of participants through the face-to-face interview. All results of the IPAQ were expressed and analyzed as metabolic equivalents per minute (MET-minutes per week).

### Statistical analysis

All values are reported as mean ± S.E.M. A paired t-test analysis was performed to identify the genes whose expression levels changed significantly in each group. Independent t-test was used to compare the mean of calorie intake and calorie expenditure between two groups.

We compared pre- and post-intervention values using the REST software and analyzed means of two groups using independent t-test. Due to the relatively small number of homozygous risk allele carriers, only dominant models were used in the genotype analysis. Graphs were made using GraphPad Prism (GraphPad Prism version 7.0 for Windows, GraphPad Software, Inc). Kolmogorov–Smirnov test was used to see if the data were normally distributed. Data were analyzed with SPSS for Windows (version 16.0, SPSS Inc., Chicago, IL, USA). A P value of < 0.05 was considered significant in all cases.

### Ethics approval and consent to participate

This study was approved by the Ethics Committee of Shahid Beheshti University of Medical Sciences (Reference Number: Ir.sbmu.nnftri.rec. 1394.22), Tehran, Iran. The schools that were involved in this study were asked permission to be part of this trial and consented for their students to participate. The details of the study were explained to students and their parents with an explanatory letter and written informed consent was obtained from both parents and students prior to joining the project.

## Results

**Fig. 1:**
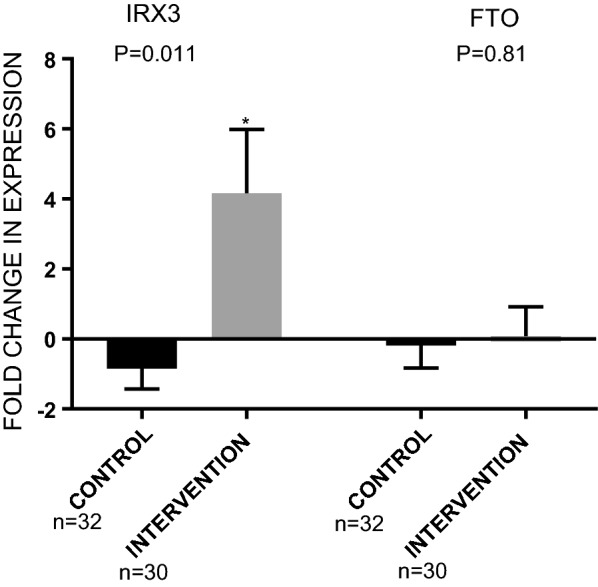
Gene expression of *IRX3* and *FTO* in blood samples of the intervention and control groups after 18 weeks. The fold change represents the ratio of the expression of the gene at the end of study to its expression at baseline. Data are presented as mean ± S.E.M. P-value was calculated using a two-tailed distribution independent t-test. *P < 0.05. As you note, gray is used for the graphs of the intervention group and black is used for the graphs of the control group

All measurement data were normally distributed (P > 0.05). No significant differences were found between two groups in terms of dietary intake physical activity at baseline. To investigate whether exposure to intensive lifestyle counseling can affect the expression of obesity-associated genes in overweight adolescent males, mRNA levels of the *FTO* and *IRX3* genes were analyzed. *IRX3* expression was significantly up-regulated in the PBMCs of the intervention group compared to baseline (P = 0.007), but remained at the same level in the control group. Moreover, the intervention group had significantly up-regulated transcripts of *IRX3* gene (P = 0.011) compared to the control group (Fig. 1). The *FTO* gene expression level did not differ significantly between the baseline and at the end of the study or between the intervention and control groups.

**Fig. 2:**
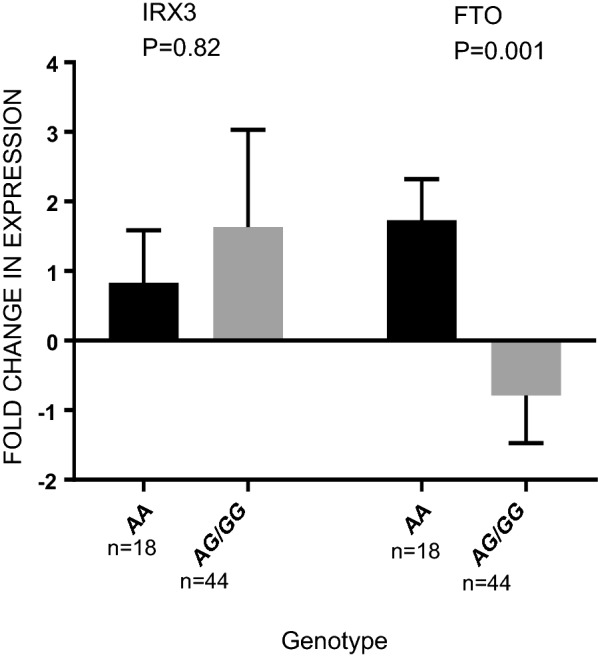
BMI-associated SNP is associated with expression of *FTO*, but not IRX3, in PBMCs. Gray is used for the graphs of the subjects with AG/GG genotype of rs9930506 and black is used for the graphs of the subjects with AA genotype. The allele of rs9930506 associated with increased BMI (risk allele) is correlated with decreased *FTO* expression and not with IRX3 expression

We next tested the relationship between SNP rs9930506 of the *FTO* gene with the change in the *FTO* and IRX3 gene expression levels, regardless of the intervention. We found significant association between the *FTO* genotype and *FTO* gene expression in PBMCs. The risk allele of rs9930506 (G) was negatively associated with change in expression of *FTO* (P = 0.001), but not *IRX3* (Fig. 2).

**Fig. 3:**
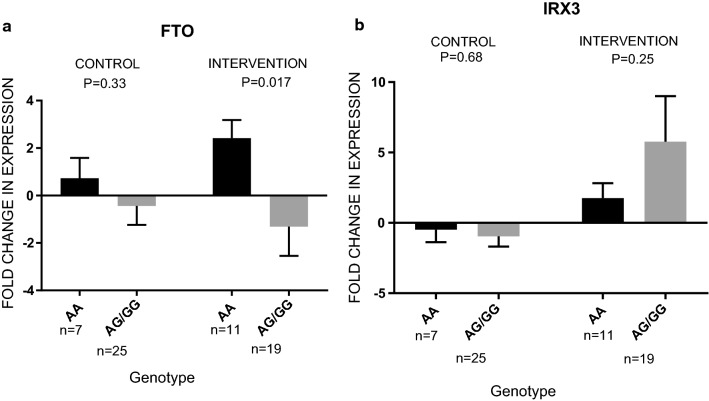
The role of *FTO* genotype on *IRX3* and *FTO* expression. Gray is used for the graphs of the subjects with AG/GG genotype of rs9930506 and black is used for the graphs of the subjects with AA genotype. *FTO* expression was up-regulated in AA carriers and down-regulated in G allele carriers in the intervention group

We also assessed the role of *FTO* genotype on change in *IRX3* and *FTO* expression for the intervention and control group separately. The frequency of AA, AG, and GG genotypes in the intervention group were 32%, 38%, 30% and in the control group were 23%, 42%, 35%, respectively. 63 percent of the intervention subjects (n = 19) and 78 percent of the control subjects (n = 25) had at least one risk allele (AG 42% and GG 35%). No significant differences were found between two groups. FTO expression was significantly down-regulated in G allele carriers in the intervention group (P = 0.017). Interestingly, the *FTO* expression was up-regulated in AA genotype carriers and down-regulated in AG/GG genotype carriers in the control group. No significant association was found, neither between *FTO* genotype and *IRX3* expression nor in *FTO* expression of control group (Fig. 3a, b). Taken together, our data showed that the effect of intervention on *FTO*, but not *IRX3*, expression depends on *FTO* genotype.

**Fig. 4:**
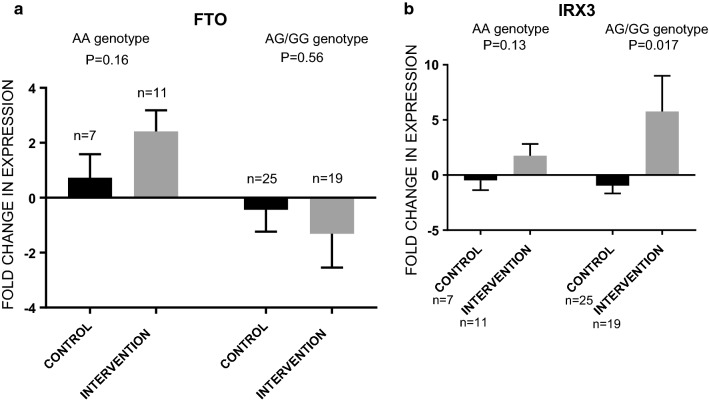
The effect of intervention genotype categories. Gray is used for the graphs of the intervention group and black is used for the graphs of the control group. In risk allele carriers, *IRX3* expression was unchanged in the control group and up-regulated in the intervention group (P = 0.017)

Next, we analysed the effect of intervention separately in AA and AG/GG carriers. The intervention group had significantly up-regulated transcripts of *IRX3* gene in the PBMCs (P = 0.017) only in risk allele carriers of the intervention group compared to risk allele carriers of the control group (Fig. 4a, b).

## Discussion

To our knowledge, the present study report has, for the first time, characterised changes in gene expression of *FTO* and *IRX3* in PBMCs after a intensive lifestyle intervention. We observed that *IRX3* expression in the intervention group was significantly up-regulated compared to baseline and compared to the control group. The intervention group had significantly up-regulated transcripts of *IRX3* gene in the PBMCs only in rs9930506 risk allele carriers of the intervention group compared to risk allele carriers of the control group. Moreover, our data showed that the effect of intervention on *FTO*, but not *IRX3*, expression depends on *FTO* genotype.

Several previous reports failed to demonstrate association of the *FTO* polymorphism with the level of *FTO* gene expression [11–13]. For example, Barton et al. investigated the relation of *FTO* gene variants to *FTO* expression and reported that *FTO* genotype was not associated with placental *FTO* expression. However, Villalobos-Comparán et al. [14] investigated the differences in relative FTO gene expression levels in human subcutaneous adipose tissue biopsies according to FTO rs9939609 genotypes under a dominant model and identified that FTO gene expression was higher for “TA/AA” risk genotypes than those with “TT” wild genotype in very obese (BMI ≥ 40 kg/m^2^) subjects. The hypothesis may be raised that a potential effect of genotype on tissue *FTO* gene expression levels may be unmasked in obesity.

Landgraf et al. [15] showed that *FTO* obesity risk variants are linked to adipocyte IRX3 increased expression in lean children, whereas it was unaffected by risk variants in obese peers. In our study, *IRX3* expression in the intervention group was significantly up-regulated only in rs9930506 risk allele carriers of the intervention group compared to risk allele carriers of the control group. It is possible that weight reduction can up-regulate *IRX3* expression. However, Smemo et al. [11] reported a direct link between IRX3 expression and regulation of body mass and composition. It has also been reported that Irx3 knockout mice were protected against obesity. Moreover, human adipocytes overexpressing IRX3 showed decreased thermogenesis [16]. Up-regulation of *IRX3* may act as a defense mechanism for protecting current body weight. Recent studies reported that the partial inhibition of hypothalamic IRX3 exacerbates obesity [11, 17]. It is possible that IRX3 acts as a modifier of lifestyle changes and the helps body adapt to different situations of calorie intake and expenditure. In line with the present study, Dankel et al. [18] found that *IRX3* was upregulated in subcutaneous adipose tissue after fat loss. These authors proposed that increased expression of homeobox transcription factors, such as *IRX3*, may improve adipose tissue functioning after its reduction. On the other hand, Ronkainen et al. [19] reported the effect of diet on IRX3 expression in adipose tissue and found that high fat diet led to 1.8-fold increase of Irx3 in Fto-knockout mice and prevented adipocytes from becoming hypertrophic after high-fat diet. Nowacka-Woszuk et al. [20] reported the importance of duration of diet regimen on the transcription of both *FTO* and *IRX3* in white adipose tissue. They reported that the transcript levels of both *FTO* and IRX3 genes decreased after 60 days and then continuously increased up to 120 days. We observed statistically significant up-regulation of IRX3 gene after about 126 days.

Recent studies examining association between *FTO* genotype and IRX3 gene expression have reported various results. For example, Ragvin et al. [21] showed that non-coding regions of the *FTO* gene affect obesity through effects on IRX3 gene transcription factors in pancreas. Moreover, Smemo et al. [11] found that obesity-associated single nucleotide polymorphisms are associated with expression of *IRX3*, but not *FTO*, in human brains. However, the present study was done on PBMCs and not adipose tissue or brain. Given the ubiquitous expression of *FTO*, the role of *FTO* in each tissue may be different from other tissues. Thus, it is expected that *FTO* gene expression in different tissues can be influenced by different factors and the different metabolic and secretion activity of these tissues.

We did not observe an effect of lifestyle changes on *FTO* gene expression in PBMCs. However, after considering *FTO* genotype, *FTO* expression was up-regulated in AA genotype carriers and down-regulated in AG/GG genotype carriers only in the intervention group. Moreover, Landgraf et al. [15] reported that the association between *FTO* risk variants and IRX3 expression was restricted to lean children and IRX3 gene expression was unaffected by *FTO* risk variants in obese children. In our study of overweight and obese adolescents, we observed that IRX3 gene expression was affected by *FTO* risk variants only in the intervention group. It was not surprising that no SNP association was seen in the control group because genes expression did not change significantly in the control group.

We suggest that the expression levels of *FTO* and IRX3 genes depend on various factors and can undergo changes in short periods of time. Gulati et al. [22] reported that *FTO* acts as a cellular sensor for some nutrients and has a role in the coupling of amino acid levels to mammalian target of rapamycin complex 1 signaling. Thus, it is possible that the expression level of *FTO* may change several times even in one day. If this hypothesis is correct, future efforts should identify all genes and signaling pathways affected by *FTO*, as well as dietary factors that affect *FTO* gene expression. These results strongly emphasize the importance of lifestyle modifications in *FTO* risk allele carriers. This study had some limitations. The extensive tissue-specific expression pattern of juvenile FTO and IRX3 genes is not possible to study in human, but it is plausible that lifestyle modifications affect the expression of FTO and *IRX3* genes in brain and adipocytes. It is plausible that the effects of lifestyle modification on the expression of these genes can be different in different tissues. Our sample was limited to adolescent boys, which make it difficult to generalize results to other age and sex groups.

## Conclusion

FTO and *IRX3* genes are suggested to have a crucial role in determining weight and BMI in adolescent boys. Lifestyle modification may exert its effects on obesity through changes in the expression level of the *FTO* and *IRX3* genes. However, *FTO* genotype plays a role in the extent of the effect of lifestyle changes on gene expression. Different alleles of the FTO gene affect the expression of the genes which might lead to a different outcome of the lifestyle modification. Further studies are needed to increase our understanding of the interaction between lifestyle, genetics, body weight and body composition.

## Additional file

**Additional file 1.** CONSORT 2010 Flow Diagram.

## Data Availability

Data are from the Interactions of Genetics, Lifestyle and Anthropometrics study, or IGLA study. Given the confined geographic area and identifying information of the dataset, data cannot be made publicly available. The Ethics Committee of the National Nutrition and Food Technology Research Institute, Tehran, Iran, specifically imposed these restrictions. Data are available from the National Nutrition and Food Technology Research Institute, Tehran, by contacting sdoaei@sbmu.ac.ir

## Abbreviations

BMI: body mass index
FTO: fat mass obesity
IRX3: Iroquois homeobox protein 3
PBMCs: peripheral blood mononuclear cells
HITT: high intensity interval training
