# Supplementary material for: Changes in FTO and IRX3 gene expression in obese and overweight male adolescents undergoing an intensive lifestyle intervention and the role of FTO genotype in this interaction
Source: J Transl Med. 2019 May 24;17:176. doi: 10.1186/s12967-019-1921-4 (PMC6534854; doi:10.1186/s12967-019-1921-4)
Supplement: Supplementary file 1 — Additional file 1. CONSORT 2010 Flow Diagram. [file 12967_2019_1921_MOESM1_ESM.doc]

**
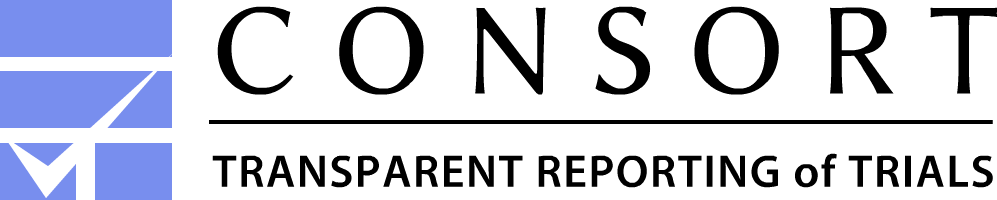
**

**CONSORT 2010 Flow Diagram**

**Allocation**

**Analysis**

**Follow-Up**

**Enrollment**

Assessed for eligibility (n=540)

Excluded (n=478)

♦  Not meeting inclusion criteria (n=294)

♦  Declined to participate (n=150)

♦  Other reasons (n=94)

Analysed (n=32)
♦ Excluded from analysis (give reasons) (n=0)

Lost to follow-up (give reasons) (n=0)

Discontinued intervention (give reasons) (n=0)

Allocated to intervention (n=32)

♦ Received allocated intervention (n=32)

♦ Did not receive allocated intervention (give reasons) (n= 0 )

Lost to follow-up (give reasons) (n=0)

Discontinued intervention (give reasons) (n=0)

Allocated to intervention (n=30)

♦ Received allocated intervention (n=30)

♦ Did not receive allocated intervention (give reasons) (n= 0 )

Analysed (n=32)
♦ Excluded from analysis (give reasons) (n=0)

Randomized (n=60)
